# Decoding Warburg's hypothesis: tumor-related mutations in the mitochondrial respiratory chain

**DOI:** 10.18632/oncotarget.6057

**Published:** 2015-10-09

**Authors:** Jose M. Garcia-Heredia, Amancio Carnero

**Affiliations:** ^1^ Instituto de Biomedicina de Sevilla (IBIS), HUVR/CSIC/Universidad de Sevilla, Sevilla, Spain; ^2^ Departamento de Bioquímica Vegetal y Biología Molecular, Facultad de Biología, Sevilla, Spain

**Keywords:** cancer, metabolic switch, Warburg's hypothesis, mitochondrial respiration, mitochondrial respiratory chain

## Abstract

Otto Warburg observed that cancer cells derived their energy from aerobic glycolysis by converting glucose to lactate. This mechanism is in opposition to the higher energy requirements of cancer cells because oxidative phosphorylation (OxPhos) produces more ATP from glucose. Warburg hypothesized that this phenomenon occurs due to the malfunction of mitochondria in cancer cells. The rediscovery of Warburg's hypothesis coincided with the discovery of mitochondrial tumor suppressor genes that may conform to Warburg's hypothesis along with the demonstrated negative impact of HIF-1 on PDH activity and the activation of HIF-1 by oncogenic signals such as activated AKT. This work summarizes the alterations in mitochondrial respiratory chain proteins that have been identified and their involvement in cancer. Also discussed is the fact that most of the mitochondrial mutations have been found in homoplasmy, indicating a positive selection during tumor evolution, thereby supporting their causal role.

## INTRODUCTION

At the beginning of the last century, Otto Warburg observed that cancer cells based their energy production on fermentation rather than oxidation, even in the presence of oxygen; therefore, these cells utilize aerobic glycolysis to derive energy from the conversion of glucose to lactate [1–3]. This metabolic switch from aerobic glucose metabolism through the respiratory chain to aerobic glycolysis is typical of cancer cells, and was named the Warburg effect in honor of its discoverer. This mechanism is in opposition to the higher energy requirements of cancer cells because oxidative phosphorylation (OxPhos) produces more ATP from glucose [4]. The large energy requirements of the constantly growing cancer cells render this phenomenon an apparent paradox. As a consequence, Warburg deduced that this change from OxPhos (normal cells) to glycolysis (cancer cells) may be a consequence of mitochondrial dysfunction, leaving cancer cells no other path for producing energy [3]. This led to the hypothesis described in the Warburg theory of cancer, which postulated that the driver of tumorigenesis is defective cellular respiration, which in turn is caused by defective mitochondrial function [1–3]. However, other possibilities exist. For example, the hypoxic conditions that are present in many solid tumors may not satisfy their requirements for oxygen, permitting the cancer cells to switch off OxPhos and favor activation of glycolysis. Oncogene activation and/or tumor suppressor gene inactivation may also produce an increase in glycolytic proteins [4].

Many researchers consider the reduction of OxPhos to be a universal feature of cancer cells. Indeed, mitochondria from tumor and normal cells are different both functionally and morphologically. However, there are reports of cancer cells with normal or even increased respiratory activity, which are in apparent contradiction to the Warburg hypothesis [5]. To explain this paradox, Smolkova et al. (2010) proposed a hypothesis that describes cancer progression as waves of differential gene expression [6]. In this model, the initial waves would be characterized by mitochondrial function suppression with stimulated glycolysis and acquisition of a typical Warburg phenotype. Subsequent waves would be characterized by the recovery of OxPhos after glutamine becomes the main source for both energy production and anabolic processes. This dependence on glutamine has been observed for many cancer cells [7]. In this manner, cancer cells can modify their metabolism to extract energy from both glutamine and glucose [8]. The Warburg effect, therefore, should be considered as only one of the changes that occur in cancer cell metabolism. For example, breast and colorectal cancer cells exhibit a Warburg phenotype due to their high glucose consumption and deregulated OxPhos [9, 10]. However, some tumor-derived cell lines (HL60, HeLa, U937) make use of mitochondrial respiration to support their growth [11]. Depending on the environment, these cells can modify their metabolism from glycolysis to respiration, showing a high level of plasticity. In addition, a Warburg effect has also been detected in normal cells. Therefore, the Warburg effect is not a cause of cancer, but a common response of cells to certain environmental conditions [12, 13].

Hypoxia is considered to be one of the main factors in the switch between glycolysis and respiration. Low O_2_ availability impairs OxPhos due to its role as a final acceptor in the mitochondrial electron transport chain (mETC) [14, 15]. The adaptation of a cell to hypoxia is highly dependent on the expression and stabilization of the protein Hypoxia Inducing Factor-a (HIF-a). The two main isoforms of this protein that are involved in the regulation of hypoxia, HIF-1a and HIF-2a, are constitutively expressed during both normoxia and hypoxia [16]. However, under normoxic conditions, HIF-a is post-translationally hydroxylated by two different proteins: Prolyl-Hydroxylase Domain (PHD) and Factor Inhibiting HIF (FIH), which marks the protein for its subsequent degradation [17, 18]. PHD has been shown to be inhibited by succinate, one of the products of the tricarboxylic acid (TCA) cycle that occurs in the mitochondrial matrix [19]. Consequently, under hypoxic conditions, there is an increase in succinate levels, PHD is inhibited and HIF-a is stabilized. In addition, ROS production also can inhibit PHD, thereby contributing to HIF-a stabilization [20]. Elevated HIF-1a levels in rapidly growing cells, such as embryonic cells and tumors, not only stimulate glycolysis, but also restrict mitochondrial respiration by inhibiting mitochondrial pyruvate dehydrogenase (PDH), thus reducing pyruvate flux into the tricarboxylic acid (TCA) cycle [21, 22]. Because the TCA cycle is the origin of NADH and succinate, which are electron donors for Complexes I and II, respectively, OxPhos is also inhibited. Adaptation to hypoxia, and also the return to normoxic conditions, must be finely regulated. In this way, when O_2_ levels return to normal, there is a decrease in succinate levels. Therefore, PHD is no longer inhibited, which leads to destabilization of HIF-a [23]. Inadequate regulation of hypoxia is an important event in the acquisition of a malignant phenotype, and demonstrates the importance of proper regulation of OxPhos.

## THE OXPHOS MACHINERY

**Figure 1:**
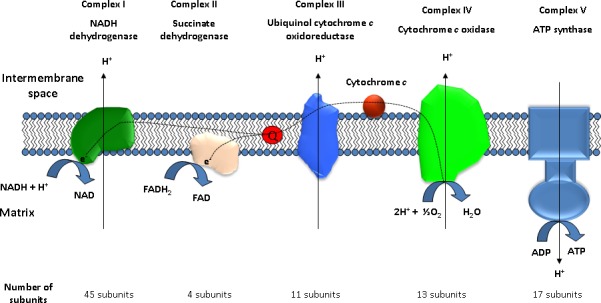
Schematic representation of the main elements of the mitochondrial respiratory chain

This finely tuned system is composed of two different and separate sets of reactions that allow ATP synthesis (Figure 1). The first is the mtETC, which is composed of four different complexes (I, II, III and IV) that are anchored to the mitochondrial inner membrane, and cytochrome c [24–26]. Together, these proteins are responsible for electron transport from NADH or succinate to oxygen, translocating protons from the matrix to the intermembrane space of mitochondria. This allows the creation of an electrochemical gradient that is exploited by the second complex, ATP synthase, to produce ATP from ADP when the protons enter again into the mitochondrial matrix through this complex [27]. Both electron transfer and proton translocation are the main effects of the mtETC, although other products are synthesized as side reactions. Approximately 1-2% of all of the oxygen that is used in respiration leaks out as superoxide (O_2_^−^) radicals and other reactive oxygen species (ROS) [28]. These highly reactive molecules must be neutralized by enzymes, such as superoxide dismutase or catalase, to diminish their negative effects [29]. However, under stressed conditions, the amount of ROS may increase and produce alterations in mitochondrial complexes due to undesirable reactions with some of the complexes [30]. In addition, ROS can also react with lipids such as cardiolipin, a mitochondrial phospholipid essential for the correct assembly and activity of complexes I, III and IV [31]. However, the long-term dramatic consequences induced by ROS are alterations in mitochondrial DNA (mtDNA).

The mitochondrial genome encodes 13 polypeptides for OxPhos and 24 genes for tRNA and rRNA [32]. It has been estimated that the susceptibility of mtDNA to damage is approximately 10-fold higher than of nuclear DNA, due to the absence of histone-protection and its direct exposure to ROS that are generated during OxPhos [33, 34]. In addition, mtDNA replication by DNA polymerase γ occurs with lower fidelity than with nuclear DNA, due to mtDNA's inferior proof-reading function [35]. It is known that oxidative damage to DNA induces two different types of transitions: T>C and G>A; therefore, it is reasonable to expect a higher frequency of these mutations in mtDNA after ROS insult [36]. Therefore, because parts of the complexes I, III, IV and V are encoded by the mitochondrial genome, damage to the mitochondrial genome can alter OxPhos and contribute to malignant transformation. Indeed, mtDNA mutations are associated with a wide variety of cancers such as thyroid, colon, ovary, breast, prostate, liver, pancreas, brain, lung and gastric carcinomas [37–39] (Tables 1 and 2). Many of the mutations in mtDNA have been detected in the D-loop, a non-coding region of the mitochondrial genome that is involved in DNA replication and RNA transcription. These mutations have been detected in a high number of tumors [38]. Furthermore, mutations in the coding regions of mtDNA have also been widely described in cancer. Approximately 600 different mutations affecting Complex I alone have been described in different cancers [38], making the analysis of each of these mutations and their contribution to cancer progression difficult. To complicate the issue even further, a normal cell can harbor multiple mtDNA mutations with no apparent abnormal phenotype. An individual cell contains multiple mitochondria, and each mitochondrion can possess up to 10 copies of mtDNA [29]. This implies that when mutations occur in mtDNA, the mutations must reach a threshold to exhibit a significant effect. Indeed, more than 70% of all tumor-specific mutations have also been detected in control populations [40]. Thus, at least 60% of all mitochondria in a cell would have a specific mutation [29]. However, many cancer-related mutations in mtDNA appear in homoplasmy (all mtDNA copies are identical), suggesting that they are positively selected during tumor evolution. This effect suggests that if a specific mutation occurs in the germline, this mtDNA modification could induce the appearance of tumors in offspring [29]. However, the lack of these mutations in tumors suggests that mtDNA mutations appear as a consequence of oxidative stress and cell division.

**Table 1:** Mutations in Complex I that are related to changes in tumorigenic properties

Subunit	Mutation	Amino acid substitution	Properties	DNA homogeneity	Tumor	Ref
mtND1	m.3308T>C	M1T	Protumorigenic	Homoplasmy	Colorectal cancer; Oncocytoma	[56]
	m.3460G>A		Protumorigenic	Homoplasmy	In vitro	[55]
	m.3571insC	Frameshift	Antitumorigenic	Homoplasmy	Oncocytoma, in vitro	[4, 55]
	m.3571insC	Frameshift	Protumorigenic	Heteroplasmy	Thyroid oncocytoma	[53]
mtND2	m.4605A>G		Protumorigenic	Homoplasmy	Head & neck squamous cell carcinomas	[33]
	m.4831G>A	G121D	Protumorigenic	Homoplasmy	Head & neck squamous cell carcinomas	[33]
	m.4776G>A	A→T	Protumorigenic	Homoplasmy	Head & neck squamous cell carcinomas	[58]
mtND3	m.10398A>G	T114A	Protumorigenic	N.D.	Breast cancer	[59]
mtND4	m.12084C>T		Protumorigenic	Homoplasmy	MDA-MB-231	[60]
mtND5	m.12418insA	Frameshift	Protumorigenic	Heteroplasmy & Homoplasmy	Colorectal cancer	[56, 61]
	m.13966A>G		Protumorigenic	N.D.	MDA-MB-231	[60]
mtND6	m.14111insC*	Frameshift	Protumorigenic	Homoplasmy	Lewis lung carcinoma	[45]
	m.14223G>A#	P25L	Protumorigenic	Homoplasmy	Lewis lung carcinoma	[45, 63, 64]
mtND4L	m.10563T>C		Protumorigenic	Homoplasmy	Colorectal cancer	[56]
	m.10695G>A	A76T	Protumorigenic	N.D.	Head & neck squamous cell carcinomas	[33]
NDUFB1		R81N	Protumorigenic	Heterozygosis	Oncocytoma	[65]
NDUFB6		E8V	Protumorigenic	Heterozygosis	Oncocytoma	[65]
NDUFA12		I134Δ	Protumorigenic	Heterozygosis	Oncocytoma	[65]

* assigned as m.13885insC; #: assigned as m.13997G>A. Both mtDNA positions correspond to mtND5 according to MITOMAP and NCBI databases, while P25L is correctly assigned to the mtND6 gene.

**Table 2:** Mutations in Complexes III, IV and V that are related to changes in tumorigenic properties

Subunit	Mutation	Amino acid substitution	Properties	DNA homogeneity	Tumor	Ref
Mt-Cyb	m.15342insT	Frameshift	Protumorigenic	Homoplasmy	Colorectal cancer	[37]
	m.15557G>A	E271K	Protumorigenic	Heteroplasmy	Thyroid oncocytoma	[53]
	Δ4-cytb	Frameshift		Heteroplasmy		[90]
	m.15642-15662del	Frameshift	Protumorigenic	N.D.	Primary bladder tumors	[91]
MTCO1	m.6124T>C	M74T	Protumorigenic	Heteroplasmy	Prostate cancer cells	[104]
	m.6267A>G	A122T	Protumorigenic	Homoplasmy & Heteroplasmy	breast, colon, pancreatic & prostate cancer cells	[103]
	m.6277A>G	G125D	N.D.	Homoplasmy	Prostate cancer cells	[105]
	m.7275T>C	S458P	N.D.	Homoplasmy	Prostate cancer cells	[105]
mtATP6	m.8860G>A		Protumorigenic	Homoplasmy	Breast cancer	[109]
	m.8993T>G	L156R	Protumorigenic	Homoplasmy	*In vitro*	[110]
	m.9176T>C	L217P	Protumorigenic	Homoplasmy	*In vitro*	[110]

Sequencing of tumor cell mtDNA has identified mutations in at least one of the 37 genes encoded by the mitochondrial genome [41]. Most of the cells that were examined exhibited decreased OxPhos, which was compensated for by an increase in glycolysis. This review will focus on proteins that are members of each of the OxPhos complexes, whose mutations have been described to be a cause, and not mere, of cancer progression due to a defect in OxPhos that can induce the Warburg phenotype (Tables 1 and 2). To understand the specific effects of a mutation, or the contribution of mitochondria to the acquisition of a malignant phenotype, cybrids have emerged as a useful strategy that can be used to analyze the role of mitochondria and their alterations in cancer progression to show that alterations other than those in nuclear DNA are involved in promoting cell malignancy. These cybrids are generated by fusing whole cells with enucleated cells (also called cytoplasts).

## COMPLEX I

This complex, also known as NADH:ubiquinone oxidoreductase, is responsible for NADH oxidation and promotes electron transfer in OxPhos. Complex I removes two electrons from NADH to facilitate the reduction of ubiquinone to ubiquinol. It is composed of 45 subunits in mammals, 14 of which constitute the core of the complex, which is sufficient for energy transduction [24]. The others are considered as accessory or supernumerary subunits. This complex is located in the mitochondrial inner membrane, with a recently solved L-shaped structure that protrudes into the matrix [42]. Complex I can be divided into two different modules: an N-module that is responsible for NADH binding and oxidation, and a Q-module that is responsible for proton pumping. Seven hydrophobic proteins of the Q-module (mtND1 to mtND6 and mtND4L) are encoded by mitochondrial DNA and are embedded in the mitochondrial inner membrane. The other 7 proteins of the core are encoded by nuclear DNA; three of them belong to the N-module (NDUFV1, NDUFV2 and NDUFS1), while the remainder belong to the Q-module (NDUFS2, NDUFS3, NDUFS7 and NDUFS8) [24]. All of the nuclear-encoded proteins are hydrophilic and are directed toward the mitochondrial matrix.

A deficiency in this complex has been connected to some human disorders, such as myopathy or neurodegenerative disorders [24, 43]. Because Complex I subunits are encoded by mtDNA or nuclear DNA (nDNA), these diseases may be attributed to mutations in at least one of the two genomes. Mutations that diminish the performance of Complex I have been described in all of the core units and in some of the supernumerary subunits, and are the causes of different disorders [44]. However, experiments with cybrids have shown that pathogenic mutations in Complex I that are involved in cancer progression are derived from mutations in mtDNA [45]. These mutations induce a change in tumor progression due to an impairment of Complex I.

Most of the mutations related to cancer tend to be homoplasmic [37, 46]. Two different types of mtDNA mutations have been described. The first type, which includes most of the described mutations, is comprised of a neutral or protumorigenic mutation that increases tumor growth. For example, some mtDNA mutations in Complex I subunits have been associated with glioblastoma, colon carcinoma and renal oncocytomas [37, 47, 48]. However, other mtDNA mutations have been associated with the arrest of tumor growth because the cells cannot acquire the advantageous Warburg profile [4]. These opposing effects of mtDNA mutations, both protumorigenic or antitumorigenic, depend on the percentage of mitochondrial heteroplasmy (more than one mtDNA genome), the effect of these mutations in Complex I subversion and the nuclear background.

The tumors that are most frequently associated with mutations in Complex I are oncocytomas [37, 49, 50]. These tumors are derived from epithelial tissue and usually appear in endocrine and exocrine organs. They are usually described as benign with low invasiveness. Histological analysis of these tumors revealed a hyperproliferation of altered mitochondria, which suggested a dysfunction that is linked to mutations in different subunits of Complex I [4, 51]. The most typical mutations related to this oncocytic phenotype have been described for the mtND1 protein, although mtND4 and mtND5 mutations, which are usually frameshift and nonsense mutations, have also been associated with this tumor [49, 52, 53]. It has been shown that when one of the subunits is mutated, the entire complex is destabilized, with lower protein levels of other subunits [31, 54]. It is likely that several subunits of Complex I are required to allow for its proper assembly and degradation of all of the subunits may occur if only one is lacking. Previous studies on 30 oncocytic thyroid samples with mtDNA mutations showed that 25 tumors exhibited at least one Complex I subunit mutation [52]. A comparative analysis between renal oncocytomas and clear cell renal cell carcinomas showed that only the oncocytomas had mutations in mtDNA genes that code for Complex I subunits [37].

Despite the clear connection between these mutations and the appearance of an oncocytic tumor, in most cases there are few studies that explain the role of a specific mutation. One reason for this lack of studies might be that the effects appear redundant, resulting in a non-functional Complex I.

### mtND1

This protein connects the hydrophilic core, where NADH is oxidized, and the hydrophobic core, which allows electron transport to ubiquinone. Therefore, any mutation here would critically affect Complex I functionality. Thus, some of the mutations described for mtND1 exhibited protumorigenic properties, while others exhibited antitumorigenic properties.

The homoplasmic mutation m.3460G>A, which has a minimal effect on Complex I activity, was introduced in tumor cells to analyze osteosarcoma progression [55]. However, there were no significant changes in cell growth, perhaps due to the nuclear background. Therefore, this mutation could be protumorigenic because the increase in ROS levels and the higher resistance to apoptosis induced. Another mutation in colorectal cancer, m.3308T>C, appears in homoplasmy, suggesting a selective growth advantage during tumor evolution [56].

However, the m.3571insC mutation, which appears frequently in oncocytomas, behaved as a tumor suppressor mutation, inhibiting tumor growth when it was tested under homoplasmic conditions [4, 53, 55]. This mutation was described in the oncocytic thyroid cell line XTC.UC1, and induces a severe alteration of the complex due to the insertion of a premature stop codon at position 101 [53]. As a consequence, these cells exhibit defects in Complex I, and, by extension, in OxPhos, that cause an imbalance in the TCA cycle, yielding a higher amount of α-ketoglutarate, which is a substrate for PHD [57]. In addition, ATP synthesis and oxygen consumption suffered a large decrease relative to control cells. As a consequence, HIF-1a is destabilized, being tumor growth arrested. Thus, it has been shown that allotypic Complex I complementation allows HIF-1a stabilization and the recovery of tumorigenic potential [4]. However, a full deficiency in Complex I results in HIF-a stabilization, suggesting that the lack of Complex I suppresses oxidative stress, while other mutations that only reduce its efficiency produce an increase in ROS production.

### mtND2

In head and neck squamous cell carcinomas, two different mutations in the mtND2 gene, m.4605A>G and m.4831G>A, were identified, and their roles in tumorigenesis were studied [33]. To accomplish this, the authors created a nuclear-encoded version of this gene (nND2) with a signal that allows for its transport to mitochondria. HeLa cells were chosen for transfection studies because these cells have no mutations in the mtND2 gene. Both mutations induced an increase in ROS production and aerobic glycolysis rate with HIF-1a stabilization, resulting in higher tumorigenicity. In addition, expression of mutated nND2 in head and neck squamous cancer cell line O19 exhibited a higher number of colonies of O19 cells that expressed wild-type nND2. Another study analyzed the mutation m.4776G>A in HeLa, OKF6 and O19 cells transfected with this mutated mtND2 [58]. Similar to the other two mutations, m.4776G>A induced HIF-1a upregulation. Xenografted tumors from HeLa cells that were stably transfected with m.4776G>A ND2 had a higher growth rate than HeLa WT cells [58]. In addition, the authors demonstrated that an increased production of pyruvate in cells expressing the mutant mtND2 was related to an increase in PDK2, a kinase that phosphorylates and inactivates Pyruvate Dehydrogenase (PDH), leading to increased ROS production and contributing to HIF-1a stabilization.

### mtND3

In this case, the mutation m.10398G>A has been shown to be associated with an increased risk of breast cancer in two different studies, one of a European American female, and the other of Jewish females [59]. This position in the mitochondrial genome is highly polymorphic, and depending on the ethnic derivation, studies concerning its role in breast cancer show an increased risk or no effect at all. This suggests again that other changes are required to promote cancer progression.

### mtND4

MDA-MB-231 cells, which are derived from human breast carcinoma, are characterized by a high metastatic potential. To analyze the possible contribution of nuclear or mitochondrial mutations to this metastatic phenotype, mitochondria were extracted from them to obtain the ρ^0^ MDA-MB-231 cell line [60]. Cybrids were obtained from this cell line using purified mitochondria from HeLa cells that were previously modified with fetal mitochondria that provided a normal respiratory function. As controls, cybrids were also generated with MDA-MB-231 mitochondria and ρ^0^ MDA-MB-231 cells. Thus, two different MDA-MB-231 cells were generated. The first can be considered to be the wild type because of the presence of the original mitochondria. The other cell line, 231mtFt, contains only normal mitochondria. As a result of this change in the mitochondrial population, 231mtFt cells exhibited a significant decrease (but not suppression) in their metastatic potentials, showing that changes in mtDNA are partially responsible for this phenotype. Two mismatch mutations were present in mtDNA from MDA-MB-231: m.12084C>T, which affects the mtND4 subunit, and m.13966A>G, which affects the mtND5 subunit. It should be noted that the observed phenotype can be the sum of both mutations, or the effect of a single one.

### mtND5

The frameshift mutation m.12418insA causes a disruption to the mtND5 subunit. This change has been detected as a heteroplasmic mutation in cells from colorectal cancer [56]. The C8T cell line, which *in vitro* resembles the heteroplasmic condition, exhibited an increase in tumorigenic properties compared to 143B, a human osteosarcoma-derived cell line, despite the lack of a functional Complex I. In addition, C9T cells, which have the m.12418insA mutation in homoplasmy, exhibited the lowest tumorigenic properties, showing that, in this case, heteroplasmic conditions constitute an advantage for tumor growth [61]. These cells have a higher level of ROS production because of impairment in OxPhos, and also a higher resistance to apoptosis that is induced by oxidative stress. In an elegant experiment, Sharma et al. demonstrated an association between Complex I dysfunction (due to mtND5 mutations) and tumorigenicity [62]. To accomplish this, they used the *Saccharomyces cerevisiae* NADH protein quinone oxidoreductase (NDI1), a single protein that mimics the function of the entire Complex I, and which is encoded in the nucleus. Despite having a non-functional Complex I, NDI1 expression restored mitochondrial functions, inducing a reversal of tumorigenic properties and a reduction of ROS production.

Disruption of the mtND5 subunit has been shown to induce oxidative stress because of an increase in ROS production that is due to Complex I dysfunction [61, 62]. This increment has been associated with AKT signaling though an increase in AKT phosphorylation and by upregulation of downstream factors such as HIF-1a, Bcl-XL and Mcl1[62]. Activation of these genes is recognized as a common event in tumorigenesis. However, homoplasmic mutations induce a higher apoptotic rate, suggesting that mtDNA mutations contribute to tumorigenesis under heteroplasmy conditions.

### mtND6

mtND6 gene mutations have been detected in Lewis lung carcinoma. Two different mutations, m.14111insC and m.14223G>A (originally assigned as m.13885insC and m.13997G>A) are associated with a higher metastatic potential [45]. The relationship between m.14111insC and its metastatic potential was demonstrated when, after exchanging mtDNA between high- and low-metastatic mouse lung carcinoma cell lines (A11 and P29, respectively), these properties were also exchanged. In addition, metastatic properties were reverted by treating cells with antioxidant agents, showing the importance of ROS in metastasis. The other mutation, m.14223G>A, has been shown to be homoplasmic and to produce higher levels of ROS. In addition, cells with mutations in mtND6 contained a non-functional Complex I and an upregulated HIF-1a, VEGF and MCL-1, all of which were associated with increased tumorigenicity.

Mice expressing mtND6 m.14223G>A exhibited a defective Complex I and symptoms of lactic acidosis, with no additional phenotypes in young individuals [63]. In fact, tissues from mice had a compensated phenotype with higher Complex I activity than that exhibited by homoplasmic m.14223G>A cells. In addition, ROS levels in mutant mice were similar to ROS levels in the WT mice. However, aged mice exhibited a higher incidence of lymphoma, although B6 mice, the nuclear donor, exhibited a frequent lymphoma rate, thus the mutation only acts by increasing this rate. In a recent paper, the authors used A/J mice as receptors of m.14223G>A because this strain has no tendency to develop lymphomas [64]. As a result, the lymphoma frequency was not increased in mice with the m.14223G>A mutation, showing that an additive effect between the nuclear background and mtDNA mutations must occur to increase the tumorigenic properties.

### mtND4L

m.10563T>C has also been described in homoplasmic conditions in colorectal cancer cells, showing that this mutation confers an advantage on the cells during cancer progression [56]. Other mutations, such as m.10695G>A, have also been shown to be associated with tumor progression [33].

### Nuclear encoded proteins

Mutations that cause a deficiency in Complex I have been identified in the 7 core proteins that are encoded by nuclear DNA and also in supernumerary subunits and assembly factors. However, there is a limited amount of data concerning the role of these mutations in tumorigenesis [44]. In oncocytic samples, the most typical tumor attributed to Complex I mutations, the 38 nuclear genes encoding the remainder of the subunits of this complex were analyzed [65]. Thus, three heterozygous modifications with possible effects on Complex I functionality were detected: the missense changes R81N in NDUFB1 and E8V in NDUFB6 and a deletion of three nucleotides erasing Ile134 in NDUFA12 [65]. These changes appeared with a higher frequency in oncocytic samples relative to control samples, suggesting a possible connection with the disease.

However, it should be noted that most of the mutations that were detected appear in heterozygosis, and with lower frequencies than with mtDNA mutations [45, 66]. This suggests that mutations in nuclear-encoded Complex I genes have a more deleterious effect with unfavorable consequences for cell viability than do mutations in mtDNA that usually appear in heteroplasmy. In fact, nDNA mutations have been linked with other diseases such as Leigh syndrome, cardiomyopathy or encephalomyopathy [24, 67].

To conclude, the role of the mutations that affect Complex I in mutagenesis is redundant. All of these mutations have similar consequences: changes in Complex I functionality that promote an increase in ROS production [68], an imbalance in the TCA cycle, inhibition of PHD and, consequently, HIF-a stabilization. A downstream transcription of hypoxia-related genes under normoxic conditions is one of the most common events in tumorigenesis.

## COMPLEX II

Complex II, or succinate dehydrogenase ubiquinone-ubiquinol reductase, is a member of the TCA cycle and is attached to the internal side of the mitochondrial inner membrane. It is the smallest complex that participates in OxPhos, containing only four subunits, all of which are encoded by nuclear DNA and are imported into the mitochondria where the complex is assembled after cofactor addition and protein folding [69]. As a member of the TCA cycle, it is involved in succinate oxidation to fumarate and is also an electron donor to ubiquinone in OxPhos, but not as a proton pump through the inner mitochondrial membrane. The two catalytic subunits are SDHA, a flavoprotein, and SDHB, an iron-sulfur protein, which are bound to the inner mitochondrial membrane through interactions with the other two components of the complex, SDHC and SDHD. Both membrane subunits constitute the heme-protein cytochrome b in this complex that promotes its interaction with ubiquinone for electron transfer in OxPhos.

A large number of mutations within the four components of this Complex have been indirectly linked to different types of tumors, although most of the existing studies do not focus on specific mutations. Mutations in this complex have been mainly associated with pheochromocytomas and paragangliomas, tumors of the parasympathetic and sympathetic nervous system that affect the adrenal gland and the carotid body [69]. Indeed, patients with these types of tumors are evaluated clinically to detect mutations in proteins of Complex II. At least 30% of all these tumors are derived from a germline mutation in one of the susceptibility genes. Mutations in the SDHC and SDHD subunits are associated with head and neck paragangliomas, while mutations in the SDHB subunit have been linked to malignant paragangliomas. Mutations in SDHA and SDHB have been detected also in gastrointestinal stromal tumors (GISTs) [70].

### SDHA

SDHA constitutes the major catalytic subunit of Complex II, with a covalently attached flavin adenine dinucleotide as a prosthetic group [71]. In addition, both enzyme substrates and physiological regulators bind to SDHA. The *Sdha* gene is composed of 15 different exons, thus rendering its analysis to detect mutations related to cancer difficult; thus it is not routinely analyzed [72]. However, 15 pathogenic mutations in SDHA have been described, most of them related to GISTs [25, 72]. Only three mutations have been described in paragangliomas and pheochromocytomas, the most common tumors that are produced by mutations in Complex II, [73]. These mutations, the biochemical analysis of which has uncovered their relationship to cancer, may have a very low incidence, with most of the mutations producing no clinical symptoms.

### SDHB

This gene has the highest mutation rate of all Complex II proteins, causing extra-adrenal paragangliomas, adrenal pheochromocytomas, head and neck paragangliomas and papillary thyroid cancer [25, 69]. Germline mutations in this protein are associated with a higher risk of developing renal cell carcinoma [74]. In addition, in the absence of KIT and PDGFRA mutations, both of which are characteristic of GISTs tumors, SDHB mutations have been related to GIST development [70]. Carriers of mutations in SDHB have a 25-40% probability of developing a tumor and a 20% probability of metastatic disease [25]. Indeed, mutated SDHB in tumors increase the risk of the appearance of malignant tumors, and have a poor prognosis. Paragangliomas and renal cell carcinomas derived from mutations in the SDHB gene had giant mitochondria in their cell cytoplasm [75].

### SDHC

SDHC constitutes a large subunit of cytochrome b and is anchored to the mitochondrial membrane. Mutations in this gene primarily produce head and neck paragangliomas, although some sympathetic paragangliomas have also been reported [76, 77]. However, mutated SDHC has been detected less frequently than mutations in SDHA or SDHB, and have a very low tendency toward malignant transformation [25].

*In vitro* experiments using NIH3T3 cells transformed with a mutated SDHC demonstrated both a higher rate of apoptosis and tumorigenicity [78]. However, it should be noted that transformed NIH3T3 had to be cultured for at least one month to increase its ability to form tumors after injection. This suggests that a selection phase occurs after cell transformation with mutant SDHC, with most of the cells dying because of the accumulation of ROS. The requirement of at least one month of survival for the cells in order for them to develop tumors suggests an SDHC-derived higher mutation rate.

### SDHD

Mutations in this protein are usually associated with benign head and neck paragangliomas, although they also have been detected in sympathetic paragangliomas and adrenal pheochromocytomas [79]. A decreased expression of SDHD has also been associated with gastric and colon carcinomas. It has been shown that if the mutation is inherited only from the father, then the carriers have a high probability of developing tumors that are derived from SDHD mutations. However, experiments with *Sdhd* knockout mice demonstrated an absence of paraganglioma or pheochromocytoma development, suggesting that other mutations are required to form tumors [80].

### SDHAF2 and FH

In addition to the mutations in the four proteins that constitute Complex II, other mutations have been described in proteins that are related to this complex. The G78R mutation in succinate dehydrogenase complex assembly factor 2 (SDHAF2) was identified in a head and neck paraganglioma [71]. SDHAF2 adds the FAD prosthetic group to SDHA, thus a defect in SDHAF2 results in a loss of Complex II function, as was demonstrated in yeast experiments [71]. As with SDHD, only mutations with a paternal origin have been associated with tumorigenesis [81]. Mutations in fumarate hydratase (FH), an enzyme in the TCA cycle that follows SDH, have been associated with multiple cutaneous leiomyomas, uterine leiomyomas and aggressive renal cell carcinomas [82].

In conclusion, mutations in Complex II result in the formation of a non-functional complex, leading to the accumulation of succinate that acts as an inhibitor of PHD. Due to the constitutive expression of HIF-a, the lack of active PHD produces HIF-a stabilization, and transcription of hypoxia-dependent genes, which is associated with a higher tumorigenicity potential. Thus, an activated HIF-a pathway has been detected in SDH-deficient tumors [80, 83, 84]. However, human breast cancer samples exhibited a low correlation with HIF-1a stabilization [85]. Nevertheless, there are no animal models that resemble the human phenotype because heterozygous animals do not develop cancer, and homozygosity is lethal [86, 87]. In addition, there are no established cell lines that are derived from Complex II-related tumors. Consequently, additional research concerning the role of Complex II in tumorigenicity, for example using cybrids, is required.

## COMPLEX III

This complex, ubiquinol-cytochrome c reductase, promotes electron transfer between ubiquinol and oxidized cytochrome c. In its native form it is a dimeric complex comprised of eleven different subunits, with only the cytochrome b subunit (cytochrome bc1 complex subunit 3) being coded by mtDNA [88]. To date, only cytochrome b has been found mutated in some diseases, and there are no mutations for nuclear-encoded genes [89].

Complex III is one of the main sites for superoxide production, thus a deficiency or malfunction at this site can increase ROS-associated damage. Therefore, unlike Complexes I and II, for which ROS production is in the mitochondrial matrix, Complex III can also release superoxide to the intermembrane space.

### Cytochrome b

Various mutations, including deletions, frameshift and missense mutations, have been described in mtDNA genes, with most of the mutations being located outside of the trans-membrane domains. It has been shown that mutations in Complex III are also associated with a non-functional Complex I because of its possible association in a respirasome supercomplex. Mutations in Complex III, such as the frameshift mutation m.15342insT, have been found also in oncocytomas[37]. The thyroid oncocytic follicular cell line XTC.UC1 contains a combined frameshift mutation in ND1 and a non-conservative substitution, m.15557G>A, in cytochrome b [53]. This mutation induced a significant decrease in complex III activity, although the heteroplasmy with wild-type alleles may be sufficient to complement the deleterious effects in these cells.

Cybrids with a 4-bp deletion in the cytochrome b gene, a mutation that has been described in Parkinson disease, exhibited a loss of oxygen consumption with ROS production under hypoxic conditions and a stabilized HIF-1a [90]. The overexpression of a 21-bp deletion in the cytochrome b gene in carcinoma cells induced an increase in ROS, lactate production and oxygen consumption. In addition, cells with this deletion have increased tumorigenic properties, being more invasive in murine and human bladder cancer models [91].

As with complex I, mutations in cytochrome b promote an increase in ROS levels. This increment can, consequently, induce stabilization of HIF-a by inhibiting PHD.

### Cytochrome c

This small, soluble protein, is localized in the intermembrane space and is attached to that space in two different pools, loosely and tightly bound, because of its interaction with, among other molecules, cardiolipin [92]. Its primary function is to act as a one-electron carrier between mitochondrial complexes III and IV. However, it also has a role as an apoptosis triggering protein [93]. After induction of apoptosis, cytochrome *c* is released from the mitochondria and then interacts with Apaf-1, thereby initiating the caspase activation cascade [94].

Although some mutations in cytochrome *c* have been described (M80A, G41S, Y48H, Y48E, Y48F, K72A, K72W, K72G, K72R, K72E and K72L) [95], none of them have been demonstrated to be associated with cancer [96, 97]. In addition, only G41S and Y48H have been observed in a human disease, thrombocytopenia, a syndrome characterized by a low number of platelets [97]. Both mutations are pro-apoptotic, and lead to an increase in the activation of programmed cell death. None of the other mutations have been detected *in vivo*; however, they all exhibit antiapoptotic properties, leading to non-efficient apoptosome activation and, consequently, cell survival [95].

With regard to post-translational modifications, four residues are phosphorylated *in vivo* (T28, Y48, S47 and Y97) [98]. Phosphorylation of one of these residues, Y48, was studied using a phosphomimetic substitution (Y48E), which demonstrated both a partial inhibition of electron transfer to complex IV and an inability to promote activation of caspases [99]. The authors suggested that cytochrome *c* in its phosphorylated state could act as an oncogenic protein because of apoptosis inhibition; to date, there are no biological data supporting this hypothesis. In addition, analysis of gastric carcinomas, colorectal carcinomas, breast ductal carcinomas, hepatocellular carcinomas, acute myelogenous leukemias and acute lymphoblastic leukemias detected no mutations in cytochrome *c* [96].

## COMPLEX IV

This complex, which is also called Cytochrome c Oxidase (C*c*O) is the final electron acceptor in the mitochondrial respiratory chain. It reduces molecular oxygen (O_2_) to water with four electrons that are transferred from four different molecules of cytochrome c [100]. This complex is composed of 13 different subunits in mammals, and the catalytic core has three mtDNA-encoded subunits (MTCO1, MTCO2 and MTCO3). The other 10 subunits, and other proteins related to the Complex IV assembly, are encoded by nDNA [101]. Nonsense mutations, deletions and heteroplasmic point mutations have been detected in the three mtDNA-encoded subunits. Regarding nuclear-encoded genes, mutations have been found more frequently in genes related to the Complex IV assembly, such as copper chaperones or proteins involved in heme biosynthesis. Most of the nuclear mutations, however, have been detected in non-tumor diseases.

### MTCO1

Mutations in MTCO1 are associated with a higher risk of developing prostate cancer, although these mutations were initially detected in colon cancer cells [56]. Missense mutations in MTCO1 have been detected in 11-12% of all prostate cancer patients [102]. Different models have been used to study some of the mutations found in the mitochondrial gene. The probable germline mutation m.6267A>G (A122T) is a recurrent mutation that appears with a higher frequency in cancer cells than in normal tissues [103]. This mutation affects a position that is critical for the interaction between MTCO1 and subunit III of Complex IV. Cybrids harboring this mutation exhibited a 50% decrease in Complex IV activity. Mitochondria with a heteroplasmic (50%) mutation, m.6124T>C (M74T) were also detected in a prostate tumor and were purified to generate pure wild-type or mutant cybrids [104]. For this mutation, the activity of MTCO1 decreased 29%, with a concomitant increase in ROS production. In addition, cybrids with this mutation exhibited faster growth in culture, decreased apoptotic rates and increased *in vivo* tumor growth. The last effect was observed when cybrid cells were injected into nude mice, producing larger tumors with cybrids that had the mutant version of MTCO1.

Two different missense mutations found in prostate cancer cells, m.6277A>G and m.7275T>C, were analyzed functionally using the purple bacterium *Rhodobacter sphaeroides*, a model for mitochondria [105]. The m.7275T>C mutation, which has a S458P substitution, resulted in no expression of the enzyme. The G125D substitution induced a decrease in Complex IV activity, with slower electron transfer and proton leakage. However, there are no data for these mutations with mitochondria in human cells.

### MTCO2 and MTCO3

Missense mutations in the MTCO2 gene are associated with an adverse prognostic impact in patients with acute myeloid leukemia [106]. In addition, other mutations in MTCO3 have been described that are associated with hepatocellular carcinoma [41].

It has been observed, that, under hypoxia conditions, gene transcription of the nuclear-encoded subunits I, IVi1 and Vb were significantly reduced, suggesting that the lack of a functional Complex IV can also contribute to HIF-a stabilization [107]. In addition, a decrease in Complex IV activity has also been associated with an increase in ROS production, which is not directly due to Complex IV but is a result of an impairment of the entire OxPhos chain [27, 104].

## COMPLEX V

This complex, ATP synthase, is not associated with mETC, but it can be considered as a member of OxPhos due to the H^+^ gradient that is established by Complexes I, II and IV that allows ATP synthesis. It can be divided into two different subunits, which are referred to as F_0_ and F_1_ particles and are connected by two additional structures. The five subunits of F_1_ are nuclear-encoded, as are most of the F_0_ subunits, with the exception of two (mtATP6, mtATP8) that are encoded by mtDNA [108].

Regarding both mitochondrial genes, it has been recently shown that mtATP6 has a higher mutation frequency than mtATP8, at least in breast cancer [109]. Indeed, one mutation in mtATP6, m.A8860A>G, was detected in all of the cases analyzed, suggesting that this substitution may increase the risk of breast cancer [109].

Two other different pathogenic mutations have been characterized in mtDNA for the ATP6 gene, m.8993T>G and m.9176T>C, and although they have not been linked with cancer, they are associated with Leigh syndrome [110]. To analyze the possible role of both mutations that were originally detected in patients with mitochondrial encephalomyopathy [111], cybrids with one of these mutations were obtained by fusing enucleated fibroblasts from patients to one of these mitochondrial mutations with HeLa cells without mitochondria. Then, the cells were injected into nude mice to determine their tumorigenicity. Both mutations provided an advantage in early stages of tumor growth due to a decreased apoptotic rate and faster cell growth. It was, however, shown that the m.8993T>G mutation produced a 60% decrease in the rate of ATP synthesis [112]. In addition, it was shown that both mutations decreased oxygen consumption. Another study analyzed the role of the m.8993T>G mutation by introducing it into PC3 prostate cancer cells, and demonstrated a 7-fold increment in its tumor formation ability [102]. Pathogenic mtATP6 mutations are associated with an upregulation of fibroblast growth factor 1 (FGF-1) and focal adhesion kinase (FAK) [113]. An additional mutation in the mtATP6 gene, m.8601A>G, has been found in early stage breast cancer, suggesting that missense mutations in this gene increase the risk of developing tumors [114].

## CONSEQUENCES OF MTDNA MUTATIONS IN THE CLINIC

The word “cancer” encompasses a large variety of diseases that are based on DNA alterations, making it difficult to establish general rules for treating different tumors. Mitochondria constitute an ideal target for clinical treatment due to its role as the energetic core of the cell. In fact, there are anti-cancer drugs (mitocans) that are directed against different mitochondrial elements such as metabolic inhibitors, ROS regulators and Hsp90 inhibitors [115]. The main objective for understanding the effects of mitochondrial mutations on cancer progression is to design medical strategies to arrest tumor evolution. This can help to avoid treatments with undesirable side effects. For example, mutations in mtND4 induce acquired chemoresistance during paclitaxel/carboplatin treatment [116], suggesting that this treatment should be avoided in patients with mtND4 mutations. In addition, treatment of tumors with heteroplasmic mutations can increase the level of mtDNA mutations [117, 118]. The high mutagenic rate of mtDNA, therefore, is justification for a preliminary study that would focus on certain properties of the tumor. Mitochondrial DNA extracted from a cancer biopsy can be used to determine the mutations present in the mtDNA, allowing the design of specific treatments against the tumor and minimizing the side effects.

**Figure 2:**
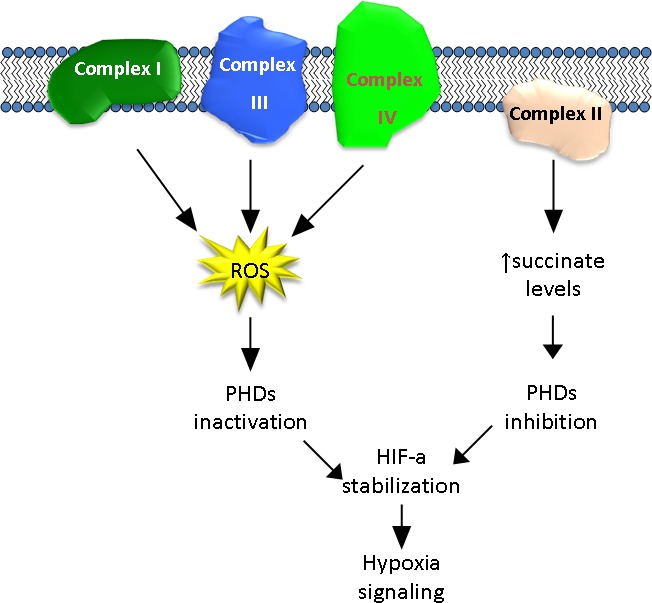
Schematic representation of the possible contributions by the different complexes of the mitochondrial respiratory chain to HIF activation

Mutations have been described in Complexes I and III, and to a lesser extent in Complexes II and IV, which promote an increase in ROS production, with HIF-a stabilization and activation of hypoxia signaling (Figure 2). This similar behavior, as a result of different mutations in different complexes, indicates that mitochondria can be considered an ideal target for the design of specific ROS-based anticancer treatments due to the large number of tumors that exhibit a Warburg profile. Mutations in Complexes I and III can be utilized in a similar manner. Although cancer cells have impaired apoptosis, the increase in ROS production can be used to induce apoptosis using prodrugs that are activated by ROS [119]. Because tumor cells have higher ROS levels than normal, this type of treatment should minimize the effects on non-tumor cells.

Regarding Complex II, although there are some reports concerning the role of this complex in the production of ROS [120–122], the main tumorigenic effect of Complex II dysfunctions will be inhibition of PHD by the accumulated succinate. In this case, the treatment should be based on cell-permeable a-ketoglutarate derivatives that restore PHD activity, thereby preventing HIF-a stabilization [123]. 2-deoxy-glucose (2-D-glucose) is a glucose analogue that induces SDH activity with a decrease in succinate levels [124], allowing PHD activation and HIF-a ubiquitination. This can be useful for mutations that occur in a partially functional Complex II.

Glycolysis, the main characteristic of the Warburg phenotype, can also be targeted using different therapeutic agents such as 2-D-glucose alone or in combination with other drugs [125]. This molecule acts by inhibiting hexokinase, which is the first enzyme of the glycolytic pathway. It has been shown that a combination of this molecule with metformin, which blocks Complex I activity, induces cell death [126, 127]. In cases of tumors with a severe impairment of Complex I, 2-D-glucose should be sufficient as treatment against these mitochondria. However, due to the variability of cancer, the treatment chosen for altered mitochondria might not be adequate to defeat the disease. Nevertheless, knowledge of the specific mtDNA mutations in a specific tumor will help to choose a better and more powerful treatment with reduced side effects.

## CONCLUDING REMARKS

Most of the described mutations affecting proteins related to OxPhos have been found to be redundant in their biological effects. Generally, a single mutation in one of the four complexes of the mtETC induces a loss of function (partial or total) that disrupts OxPhos balance. The loss of OxPhos balance usually leads to lower O_2_ consumption [53, 57, 90, 105, 110], with some exceptions, such as a 21-bp deletion in cytochrome b [91]. This decrease is also correlated with a decrease in mitochondrial membrane potential, which leads to the dysfunction of the entire organelle [26, 31, 32, 53]. When this occurs, ROS and/or succinate levels rise, inhibiting PHD, promoting HIF-a stabilization, and inducing a pseudo-hypoxic response under normoxia conditions. However, it should be noted that these mutations occur in the gene-coding sequence, but a mutation in a non-coding region, related to transcription, may have similar effects in tumor development because the lack of one of the proteins will result in a non-functional complex. In breast cancer cells, proteins of all of the complexes exhibit reduced expression from both nDNA or mtDNA [85, 128]. This suggests that we also need to consider other regions from either the nuclear or the mitochondrial genome that could be involved in gene transcription. In addition, it must be considered that the phenotype that is attributed to a specific mtDNA mutation can add to other mutations, promoting a greater tumorigenic effect. Thus, the pancreatic cancer cells lines CFPAC-1 and CAPAN-2 exhibited resistance to anticancer drugs because of their mutations in different mtDNA proteins of the different complexes of OxPhos [129].

As has been discussed in this review, most of the mitochondrial mutations have been observed in homoplasmy, showing a positive selection during tumor evolution. These mutations appear to be the cause of unbalanced OxPhos regulation through non-adequate hypoxia regulation or increase in ROS due to stressed conditions. The described mutations in proteins from Complex I to Complex IV (with the exception of cytochrome *c*) induce an increase in ROS production leading to HIF-a stabilization (Complexes I, III and IV), or directly induce HIF-a stabilization through inhibition of PHD (Complex II). Taken together, these data suggest that mitochondrial mutations could be the origin of the Warburg phenotype by way of HIF activation.
